# Is pseudoneurotic schizophrenia still a clinically relevant concept?

**DOI:** 10.1192/j.eurpsy.2026.12149

**Published:** 2026-07-31

**Authors:** N. Nikolova, A. Bogdanovska Toskikj, E. Miceva Velickoska, E. Canevska, L. Novotni

**Affiliations:** 1 PHI University Clinic of Psychiatry; 2 Medical Faculty, Skopje, North Macedonia

## Abstract

**Introduction:**

Pseudoneurotic schizophrenia described patients with pervasive “neurotic” symptoms (anxiety, dissociation, obsessions, somatic complaints) alongside subtle disturbances of thought-form and affect regulation that concealed an underlying psychotic process. This term, similar to concepts like “borderline”, “latent”, or “ambulatory” schizophrenia, fell out of use and was absorbed into schizotypal and borderline personality disorder. However, an important clinical subset remains poorly captured by these labels, a gap that has been attempted to be filled with schizophrenia spectrum and other proposed categories such as schizo-obsessive disorder.

**Objectives:**

To evaluate the clinical relevance of “pseudoneurotic schizophrenia”.

**Methods:**

A non-systematic literature review (keywords: pseudoneurotic schizophrenia; schizo-obsessive) was conducted, and a single case report is presented.

**Results:**

A 29-year-old man with complaints of severe akathisia-like inner restlessness without overt manifestation of anxiety, unusual bodily sensations (skin burning, tingling/stinging, “licking,” head pressure), dissociation, intrusive ego-dystonic obsessions with bizarre content, insomnia, and anhedonia. He was admitted to the day hospital where examination revealed multimodal hallucinations, somatic/persecutory/guilt delusions, thought interference and passivity phenomena, and a mild formal thought disorder. On admission, BPRS score was 65, and PANSS score was 90. MRI and EEG results were unremarkable. He reported the onset of psychiatric symptoms 10 years ago, and 3 years of persistent symptoms with preserved occupational functioning. History included 2 clearly delineated psychotic episodes with good antipsychotic response. Between episodes, he exhibited pervasive anxiety, marked inner restlessness, somatic complaints, intrusive obsessions, and atypical cutaneous/cenesthetic sensations, and was diagnosed with F41 and F60 and treated with SSRIs, with limited benefit. After antipsychotic treatment with partial remission (BPRS=38, PANSS=64), delusional ideation attenuated; reality testing was intact, and no formal thought disorder was elicited. Obsessive thoughts, burning cutaneous sensations, diffuse anxiety, and akathisia-like inner restlessness persisted with reduced intensity, and occupational functioning was preserved.

**Conclusions:**

A subset of patients who present with anxiety, obsessive-compulsive, and mood symptoms may be masking a psychotic process. Careful assessment of neurotic symptoms and consideration of the “pseudoneurotic” concept are essential to avoid personality/anxiety mislabeling and delayed antipsychotic treatment. These cases may indicate progression to schizophrenia. This suggests that a clinically important phenotype remains inadequately captured by current DSM-5/ICD-11 categories and retains contemporary relevance within a dimensional schizophrenia-spectrum view.

**Disclosure of Interest:**

None Declared

